# A pragmatic cluster randomized clinical trial of diabetes prevention strategies for women with gestational diabetes: design and rationale of the Gestational Diabetes’ Effects on Moms (GEM) study

**DOI:** 10.1186/1471-2393-14-21

**Published:** 2014-01-15

**Authors:** Assiamira Ferrara, Monique M Hedderson, Cheryl L Albright, Susan D Brown, Samantha F Ehrlich, Bette J Caan, Barbara Sternfeld, Nancy P Gordon, Julie A Schmittdiel, Erica P Gunderson, Ashley A Mevi, Ai-Lin Tsai, Jenny Ching, Yvonne Crites, Charles P Quesenberry

**Affiliations:** 1 Division of Research, Kaiser Permanente Northern California, 2000 Broadway, Oakland, CA, USA; 2 School of Nursing and Dental Hygiene, University of Hawaii at Manoa, Honolulu, HI, USA; 3 Division of Perinatology, Department of Obstetrics and Gynecology, Kaiser Permanente Medical Center, Santa Clara, CA, USA

**Keywords:** GDM, Diabetes prevention, Cluster randomized clinical trial, Lifestyle intervention, Comparative effectiveness

## Abstract

**Background:**

Women with gestational diabetes (GDM) are at high risk of developing diabetes later in life. After a GDM diagnosis, women receive prenatal care to control their blood glucose levels via diet, physical activity and medications. Continuing such lifestyle skills into early motherhood may reduce the risk of diabetes in this high risk population. In the *Gestational Diabetes’ Effects on Moms (GEM) study,* we are evaluating the comparative effectiveness of diabetes prevention strategies for weight management designed for pregnant/postpartum women with GDM and delivered at the health system level.

**Methods/Design:**

The GEM study is a pragmatic cluster randomized clinical trial of 44 medical facilities at Kaiser Permanente Northern California randomly assigned to either the intervention or usual care conditions, that includes 2,320 women with a GDM diagnosis between March 27, 2011 and March 30, 2012. A Diabetes Prevention Program-derived print/telephone lifestyle intervention of 13 telephonic sessions tailored to pregnant/postpartum women was developed. The effectiveness of this intervention added to usual care is to be compared to usual care practices alone, which includes two pages of printed lifestyle recommendations sent to postpartum women via mail. Primary outcomes include the proportion of women who reach a postpartum weight goal and total weight change. Secondary outcomes include postpartum glycemia, blood pressure, depression, percent of calories from fat, total caloric intake and physical activity levels. Data were collected through electronic medical records and surveys at baseline (soon after GDM diagnosis), 6 weeks (range 2 to 11 weeks), 6 months (range 12 to 34 weeks) and 12 months postpartum (range 35 to 64 weeks).

**Discussion:**

There is a need for evidence regarding the effectiveness of lifestyle modification for the prevention of diabetes in women with GDM, as well as confirmation that a diabetes prevention program delivered at the health system level is able to successfully reach this population. Given the use of a telephonic case management model, our Diabetes Prevention Program-derived print/telephone intervention has the potential to be adopted in other settings and to inform policies to promote the prevention of diabetes among women with GDM.

**Trial registration:**

Clinical Trials.gov number, NCT01344278.

## Background

Gestational diabetes (GDM) is defined as carbohydrate intolerance with onset or recognition during pregnancy [1]. GDM affects 7-14% of the pregnancies in the U.S. and the prevalence has increased 30-100% during the last decade [2]. A meta-analysis [3] of 20 studies reported that among women with GDM, the risk of developing diabetes postpartum was seven times higher than that in women with normoglycemic pregnancies. The Diabetes Prevention Program [4] (DPP) demonstrated that among people with impaired glucose tolerance, intensive lifestyle intervention reduced the risk of developing diabetes by 58% in parous women with or without a history of GDM whose pregnancies were approximately 12 years before enrollment [5]. Yet postpartum quality of care in women with GDM (e.g., glucose testing, counseling on lifestyle factors and weight management) is suboptimal [6,7]. Young women diagnosed with GDM often receive prenatal care from a team of providers in order to control blood glucose levels via diet, physical activity and medications. This focus on healthy practices may coincide with increased motivation for healthy behavior change out of concern for the infant. Continuing and adopting such lifestyle skills into early motherhood may reduce the risk of diabetes later in life in this high risk population.

We previously demonstrated the feasibility of a pregnancy and postpartum print/telephone-based lifestyle intervention based on the DPP curriculum [8]. The next step was to implement this intervention at the health system level to evaluate its translation into clinical practice. This article describes a cluster randomized intervention trial conducted among the 44 medical facilities of Kaiser Permanente Northern California (KPNC) that includes 2,320 pregnant women with GDM identified during a 12-month period. A cluster randomized trial design was selected because KPNC has a centralized print/telephone-based case management system for all pregnancies complicated by GDM across the 44 medical facilities [9]. By adapting our intervention for use in 22 randomly selected KPNC medical facilities, we can compare its effectiveness for weight management to KPNC’s usual care practices for women with GDM.

### Research goals

The primary research goal is to compare the effectiveness of each intervention strategy (print/telephone-based case management alone, i.e. usual care, or a DPP-derived print/telephone lifestyle intervention in addition to usual care) in helping women with GDM to: a) reach pregravid weight if normal weight [body mass index (BMI < 25.0 kg/m^2^)] before pregnancy; or b) achieve a 5% reduction from their pregravid weight if overweight or obese (BMI ≥ 25.0 kg/m^2^) before pregnancy.

Secondary research goals include assessing differences between the two treatment conditions in postpartum glycemia, blood pressure, depression, percent of calories from fat, total caloric intake and levels of physical activity.

Tertiary research goals include assessing whether the effectiveness of the intervention is consistent across women’s characteristics (specifically, race/ethnicity, pregravid BMI, parity, education and health literacy).

Finally, we will determine the cost-effectiveness of the lifestyle intervention and perform an evaluation according to the Reach, Efficacy, Adoption, Implementation, and Maintenance (RE-AIM) framework to assess the strengths and weaknesses of the intervention and, potentially, guide implementation into the health system [10].

## Methods/Design

### Clinical setting and usual care for women with GDM

KPNC is an integrated health care delivery system with more than 3 million members. At the start of the trial (March 2011), KPNC was comprised of 44 medical facilities and 14 delivery hospitals managing about 33,000 births per year. KPNC membership represents about 30% of the geographic area served, and closely approximates the demographic characteristics of the surrounding population [11].

In this setting, 50-g, 1-h oral glucose tolerance tests (OGTT) are performed at KPNC clinical laboratories to screen for GDM; 96% of KPNC pregnancies are screened [2]. If the screening test is abnormal, a diagnostic 100-g, 3-h OGTT is performed [12]. GDM is diagnosed if 2 or more of the 4 plasma glucose values obtained during a 100-g, 3-h OGTT meet or exceed the plasma glucose thresholds defined by Carpenter and Coustan [12,13].

In addition to the care provided by their obstetricians, all women with GDM receive supplemental print/telephone-based care from the Regional Perinatal Service Center (RPSC). The RPSC provides nurse case management [9]; it is staffed by 30 nurses and 2 dietitians who offer telephone counseling on glucose monitoring and control, diet and physical activity during pregnancy. No counseling is offered that specifically targets appropriate gestational weight gain.

At 6 weeks postpartum, the RPSC sends a reminder letter for a 75-gram OGTT to test for diabetes, as well as printed materials on healthy lifestyle encouraging attaining a healthy BMI, participating in 30 minutes of physical activity a day, and healthy eating. Women are advised to have their blood glucose level tested once a year. If the postpartum test results indicate pre-diabetes [14], the woman is sent printed materials explaining her increased risk of diabetes, how to prevent diabetes and how to sign up for educational classes on pre-diabetes offered at her medical facility. If the postpartum test results indicate that a woman has diabetes, she is asked to repeat the 75-gram OGTT and to make an appointment with her health care provider.

### Eligibility and exclusion criteria

All 44 KPNC medical facilities serviced by the RPSC are included. Pregnant women receiving medical care at the 44 medical facilities with a diagnosis of GDM [13] between March 27, 2011 and March 30, 2012, and who are 18 years of age or older, are potentially eligible (n = 2,480). Women are excluded if they had a neonatal loss (n =18) or did not have any telephone contact with the RPSC during pregnancy (n = 142), leaving 2,320 eligible women. The postpartum telephonic intervention is introduced as a new program being offered by the RPSC to new mothers with a GDM history; therefore, contact with the RPSC and willingness to participate in a case management program for GDM offered by the health system during pregnancy is required.

### Randomization

Given significant variation in the expected number of GDM cases per medical facility in a 12-month period (range: 3 – 247; mean = 57.5, sd = 50.3), randomization is blocked on medical facility size (i.e., expected number of annual cases of GDM: <25, 25–74, ≥75). We implemented a restricted randomization scheme [15], whereby we ensured an acceptable level of between–condition balance in expected racial/ethnic distributions and in number of women contacted for recruitment by an unrelated observational GDM study that was ongoing at the time of GEM study randomization. This was achieved by eliminating all allocations that did not meet specified balance criteria (i.e., maximum between-condition relative difference) from among all possible treatment allocations for the 44 medical facilities. After considering a variety of balance criteria scenarios, we chose a 20% within stratum and 10% overall balance criteria for each racial/ethnic group, and a 15% between condition balance on the expected number of GDM cases with recruitment attempts by the observational study. These criteria resulted in 44,631,615 acceptable allocations and good performance in terms of pairwise medical facility frequencies, with 95% of all clinic pairs (total of 946 pairs) randomized to the same condition with probabilities of 40% to 60% (designs resulting in many instances of cluster pairs never/rarely or always/often randomized to the same arm threaten study validity) [15].

With treatment conditions for enumerated allocations labeled as groups A and B, one allocation was randomly chosen via a random number generator, with the seed supplied by a non-study staff person (SAS® software function RANUNI) [16]. At a separate time and location, via a flip of a coin, another non-study staff person assigned group A and B to intervention/usual care groups, witnessed by the GEM study biostatistician (C.P.Q.).

### Blinding

All study investigators, biostatistician, data collectors (study interviewers and medical assistants who measure weight and blood pressure at the medical facilities), health care providers, and RPSC staff are blinded as to group assignment. Treatment group assignment is only known by the programmer of the study database.

### Usual care

Women receiving health care at KPNC medical facilities randomized to the usual care condition receive the standard telephone calls from the RPSC during pregnancy, as well as the RPSC’s postpartum printed educational materials previously described.

### Lifestyle intervention

In addition to usual care, women from the medical facilities assigned to the intervention condition receive a print/telephone-based lifestyle program, similar to the DPP curriculum, called “Getting in Balance” (GIB). The program is delivered on behalf of the RPSC in English and Spanish; sessions requiring other languages were delivered through a KPNC medical interpreter service. Following the design of the DPP [17], the program provides specific diet, physical activity and weight goals. Methods to achieve those goals are individualized to women’s preferences, resources, and cultural context using motivational interviewing and theoretical constructs derived from social cognitive theory (SCT) [18] and the Transtheoretical Model (TTM) [19], which have been used extensively in home-based and self-management programs [19,20]. Thus, the counseling approach addresses stages of change [21,22], self-efficacy [20,23], and social support for lifestyle behaviors [24].

The intervention consists of three phases: pregnancy phase I, early postpartum phase II, and late postpartum phase III (Table 1).

**Table 1:** Components of the getting in balance intervention: the Gestational Diabetes’ Effects on Moms (GEM) study

**Phase**	**Component**	**Topic**	**Modality**
Pregnancy phase I	Gestational weight gain letter	Goals for gestational weight gain tailored to pregravid BMI and current weight gain trajectory	Mail
Early postpartum phase II	Session 1	Intervention overview; healthy eating	Telephone
	Session 2	Dietary fat	Telephone
	Session 3	Low-fat healthy eating	Telephone
	Session 4	Physical activity	Telephone
	Session 5	Handling challenging feelings and triggers	Telephone
	Session 6	Healthy eating for weight loss	Telephone
	Session 7	Healthy eating out	Telephone
	Session 8	Managing setbacks	Telephone
	Session 9	Physical activity	Telephone
	Session 10	Healthy eating during social activities	Telephone
	Session 11	Managing stress	Telephone
	Session 12	Negative thoughts	Telephone
	Session 13	Staying motivated; relapse prevention	Telephone
Late postpartum phase III	Newsletter 1	Behavioral strategies (e.g., self-monitoring; problem solving)	Mail
	Newsletter 2	Dietary tips; stress management; social support	Mail
	Newsletter 3	Physical activity and dietary tips; behavioral strategies	Mail

### Pregnancy phase I

The target for the lifestyle program during pregnancy is to help women adhere to Institute of Medicine (IOM) guidelines for gestational weight gain [25]. Immediately following diagnosis with GDM, women are mailed a letter specifying a goal for total gestational weight gain by the end of pregnancy. The goal is tailored to women’s pregravid BMI and gestational weight gain trajectory (i.e., amount of weight gained relative to weeks of gestation). Since this population is at increased risk for having a larger than average infant and developing diabetes later in life, women who were normal weight (BMI 18.5-24.9 kg/m^2^), overweight (25.0-29.9 kg/m^2^), or obese (>30.0 kg/m^2^) before pregnancy are advised not to exceed the lower limit of IOM recommendations for their pregravid BMI (25, 15 and 11 lbs., respectively); women who were underweight (pregravid BMI < 18.5 kg/m^2^) are advised to gain at the midpoint of the IOM recommendations (34 lbs.) [25]. The letter explains that GDM increases diabetes risk, describes how weight management can aid in prevention, and provides healthy eating and physical activity tips to help women achieve their gestational weight gain goal.

### Early postpartum phase II

The primary target of the intervention in the early postpartum period is to help women lose the weight they gained during pregnancy if they were normal weight before pregnancy (BMI <25.0 kg/m^2^), or lose an additional 5% of their pregravid body weight if they were overweight or obese before pregnancy (BMI ≥25.0 kg/m^2^). At two weeks postpartum, women are mailed a workbook of 13 sessions through which they were guided via 30-minute (Session 1) and 15-minute telephone calls (Sessions 2–13). They also receive a fat gram and calorie counter booklet, diaries to self-monitor weight, diet, and physical activity, self-addressed stamped envelopes to return diaries to lifestyle coaches, and brochures with postpartum physical activity and healthy eating tips. Women are also referred to an interactive program-specific website. Session 1 is scheduled to occur at approximately 6 weeks postpartum, with weekly sessions occurring up to 6 months postpartum.

Women are encouraged to set weekly goals for daily intake of fat (in grams) and calories (for women not achieving their weight loss goals by monitoring fat alone). Women are encouraged to gradually work up to 150 minutes of moderate to vigorous intensity physical activity per week, starting with short bouts and gradually increasing frequency and duration. Women are encouraged to self-monitor weight, dietary intake and physical activity using diaries. Women are also encouraged to breastfeed and given information on when specific foods should be introduced to their infants.

### Late postpartum phase III

The target of the intervention in the late postpartum period is to foster women’s independent maintenance of weight loss via previously adopted lifestyle behaviors. Women are sent 3 maintenance letters between 33 and 52 weeks postpartum, encouraging active problem-solving of barriers after contact with the lifestyle coaches has ended. Letters are accompanied by diaries to encourage continued self-monitoring.

### Lifestyle coaches

Bilingual (English/Spanish) coaches are located at the RPSC. They were jointly hired by the RPSC and study investigators, but paid with study funds. Coaches are dietitians trained in physical activity, social cognitive strategies, and motivational interviewing techniques to promote health behavior change with an emphasis on tailoring recommendations to meet the needs of diverse women. Ongoing training sessions are conducted by experts at KPNC and the University of Hawaii who trained coaches as a group and as individuals using an intervention protocol, case illustrations, teleconferences, and role play. Whenever possible, women interact with the same coach to promote continuity of care.

### Intervention fidelity

Intervention fidelity is systematically assessed across multiple dimensions [26]. The trial design offers women a standard “dose,” or number, duration, frequency, and format of intervention contacts. Delivery is further standardized through written protocols, scripts, and program materials. Lifestyle coaches document process measures, e.g., session attendance, in an electronic tracking system. Adherence to the intervention protocol is monitored through process measures, audiotaped sessions, and regular group and individual supervision.

### Outcomes

*Primary outcomes* include the proportion of women who reach their postpartum weight goal and the amount of total weight change. *Secondary outcomes* include postpartum glycemia, blood pressure, depression score, percent of calories from fat, total daily caloric intake and physical activity levels. Study outcomes were collected at baseline (shortly after diagnosis with GDM) and at or near 6 weeks (range 2 to 11 weeks), 6 months (range 12 to 34 weeks) and 12 months (range 35 to 64 weeks) postpartum via two sources: 1) the electronic medical record (EMR) and 2) surveys (Table 2).

**Table 2:** Overview of data collection: the Gestational Diabetes’ Effects on Moms (GEM) study

**Domain**	**Primary data source**	**Pregnancy (Baseline)**	**6 weeks Postpartum (range 2 to 11 weeks)**	**6 months Postpartum (range 12 to 34 weeks)**	**12 months Postpartum (range 35 to 64 weeks)**
**Demographics**					
Age, race/ethnicity	EMR/Survey	X	X		
Education, country of origin, household income and size, and ethnic identity [27]	Survey	X	X		
Marital status, employment	Survey	X	X	X	X
Smoking, alcohol use, sleep	Survey	X	X	X	X
Pregravid weight and height	EMR/Survey	X			
New pregnancy	EMR/Survey			X	X
**Outcomes**					
Weight	EMR	X	X	X	X
Blood glucose	EMR	X	X	X	X
Blood pressure	EMR	X	X	X	X
Depression [28]	Survey	X	X	X	X
Fat intake by a screener [29]	Survey	X	X	X	X
Caloric intake and percent of calories from fat by Block FFQ [30]	Survey	X		X	X
Physical activity by Active Australia Survey [31]	Survey	X	X	X	X
Physical Activity by Pregnancy Physical Activity Questionnaire [32]	Survey	X		X	
**Health & Medical Care**					
Perceived health	Survey	X	X	X	X
Pregnancy and postpartum care received	Survey		X		
Medical history	EMR/Survey	X	X		
Reproductive history	Survey	X			
Family history of diabetes and GDM	Survey	X			
Medication use	EMR/Survey	X	X	X	X
Gestational weight gain	EMR				
Infant feeding [33]	Survey		X	X	X
Medical visits	EMR/Survey				X
Health literacy [34]	Survey	X			
Use of contraceptives	Survey		X	X	X
Adverse events	EMR/Survey			X	X
Health status for cost evaluation [35]	Survey		X	X	X
Heath service utilization and cost	EMR/Survey		X	X	X
**Psychosocial**					
Stage of change [21,22], Self-efficacy [20,23] and Social support [24]	Survey	X		X	
Perceived stress [36]	Survey	X		X	
Diabetes risk perception/knowledge [37]	Survey	X		X	

Weight, height, blood pressure, and blood glucose are measured in conjunction with routine care during pregnancy and throughout the 12-month postpartum period. Weight and blood pressure are measured at every health system encounter, since BMI and blood pressure are vital signs. Pregravid BMI is obtained from the EMR and calculated from weight measured prior to pregnancy (60.0%), from weight measured before 10 weeks of gestation (26.0%), from self-reported pregravid weight at the time of the first prenatal clinic visit before 10 weeks of gestation (10.5%), or self-reported at the baseline survey (3.5%). To determine the glucose tolerance status during the 12 months postpartum, the results of fasting glucose tests, 75-g oral glucose tolerance tests and hemoglobin A1c (glycated hemoglobin) are collected. All glucose and A1c tests are performed at the KPNC regional laboratory.

During pregnancy, the vast majority of KPNC patients with GDM (97%) have a clinic visit at their medical facility within 2 weeks of the diagnostic test for GDM, at which measurements of weight and blood pressure are taken. Postpartum clinical data are collected from the EMR at or near 6 weeks (range 2 to 11 weeks), 6 months (range 12 to 34 weeks) and 12 months (range 35 to 64 weeks); if a woman had multiple measurements of the outcome of interest in the range specified for a given time point, the value closest to the anchor (e.g., 6 weeks, 6 months and 12 months postpartum) is selected. KPNC patients with GDM receive a letter from the RPSC reminding them to attend a check-up at 6 weeks postpartum that includes measurements of weight, blood pressure and blood glucose. At 6 months and 12 months postpartum, the RPSC also sends a letter, for study purposes, reminding women to attend health check-ups for weight, blood pressure and blood glucose. Women also receive a telephone call reminding them to attend the postpartum health check-up. If a woman states that she is not able to attend the postpartum health check-up, she is asked to report her current weight.

To obtain data for the secondary outcomes of depressive symptoms, percent of calories from fat, total daily caloric intake, and physical activity, we also conduct surveys (Table 2). The baseline survey is conducted shortly after the diagnosis of GDM, in two phases. The first phase is administered by computer-assisted telephone interview (CATI) and includes brief questionnaires on dietary fat intake and physical activity. The second phase of the baseline survey is administered by mail and includes more detailed questionnaires on diet and physical activity. Follow-up surveys at approximately 6 weeks, 6 months, and 12 months postpartum are administered via CATI, online through a secure study website, or mail, based on participant preference. The 6-month survey is done in two phases, similar to the baseline survey. All survey modalities are available in English and Spanish.

Dietary fat intake is assessed briefly using the Block Fat Screener [29], and in detail using a validated semi-quantitative food frequency questionnaire [30] modified to accommodate the diverse dietary habits of the multiethnic cohort of GEM participants. Physical activity is assessed briefly using the Active Australia Survey [31] and in more detail using a modified version of the Pregnancy Physical Activity Questionnaire [32]. Depressive symptoms are assessed using the Patient Health Questionnaire 8 (PHQ-8) [28].

### Recruitment and retention strategies

Women are reimbursed (by gift cards) for time spent completing surveys: $40 for the first portion of the baseline survey; $50 each for the second portion of the baseline survey, the 6 week postpartum survey, and both portions of the 6 month postpartum survey; and $70 for the 12 month postpartum survey. Checks for $40 are given for weight and blood pressure assessments at 6 and 12 months postpartum. No reimbursements are provided for participating in the intervention.

Of the 2,320 eligible women, 1,706 (73.5%) completed the baseline survey during pregnancy and 1,643 (70.8%) completed the survey at 6 weeks postpartum; 1,794 (77.3%) completed either the baseline survey or 6 weeks postpartum survey. There are no meaningful differences in the recruitment rates between treatment conditions (77.7% and 77.0%, respectively). Overall, responders and non-responders are very similar in regards to several characteristics, except that responders are slightly more likely to be Non-Hispanic White (20% vs. 26%) (Table 3).

**Table 3:** Characteristics of survey responders and non-responders: the Gestational Diabetes Effects on Moms (GEM) study

	**All Sample**	**Non-responders**	**Responders**	**P-value**
	**N = 2,320**	**N = 526 (22.7%)**	**N = 1,794 (77.3%)**	
Age, years				0.21
18–24	119 (5.13)	33 (6.3)	86 (4.8)	
25–29	536 (23.1)	114 (21.7)	422 (23.5)	
30–34	863 (37.2)	184 (35.0)	679 (37.9)	
35–50	802 (34.6)	195 (37.1)	607 (33.8)	
Race/ethnicity				0.003
African American	106 (4.6)	28 (5.3)	78 (4.4)	
Asian	953 (41.1)	231 (43.9)	722 (40.3)	
Pacific Islander	38 (1.6)	13 (2.5)	25 (1.4)	
Hispanic	520 (22.4)	122 (23.2)	398 (22.2)	
Non-hispanic white	580 (25.0)	105 (20.0)	475 (26.5)	
Other	43 (1.9)	16 (3.0)	27 (1.5)	
Multiracial/Multiethnic	75 (3.2)	11 (2.1)	64 (3.6)	
Missing	5 (0.2)	0 (0.0)	5 (0.3)	
Parity				0.88
0	964 (41.6)	210 (39.9)	754 (42.0)	
1	769 (33.1)	158 (30.0)	611 (34.1)	
2	334 (14.4)	75 (14.3)	259 (14.4)	
≥3	208 (9.0)	46 (8.8)	162 (9.0)	
Unknown	45 (1.9)	37 (7.0)	8 (0.5)	
Pregravid BMI, kg/m^2^				0.11
<20.0	120 (5.2)	30 (5.7)	90 (5.0)	
20.0–24.9	667 (28.8)	163 (31.0)	504 (28.1)	
25.0–29.9	671 (28.9)	148 (28.1)	523 (29.2)	
30.0–34.9	417 (18.0)	76 (14.5)	341 (19.0)	
≥35.0	405 (17.5)	80 (15.2)	325 (18.1)	
Missing	40 (1.7)	29 (5.5)	11 (0.6)	
Gestational age at GDM diagnosis, weeks				0.75
5.6–13.9	286 (12.3)	61 (11.6)	225 (12.5)	
14.0–27.9	1092 (47.1)	245 (46.6)	847 (47.2)	
28.0–37.3	942 (40.6)	220 (41.8)	722 (40.3)	
100-g, 3-h OGTT glucose levels, mg/dl	*Mean (SD)*	*Mean (SD)*	*Mean (SD)*	
Fasting	91.3 (12.5)	90.4 (12.0)	91.6 (12.6)	0.05
1-hour	198.7 (25.2)	200.4 (25.0)	198.2 (25.3)	0.08
2-hour	176.9 (26.6)	178.1 (27.8)	176.5 (26.2)	0.24
3-hour	129.2 (32.9)	130.0 (34.1)	128.9 (32.5)	0.53
Systolic blood pressure, mmHg	114.3 (13.0)	114.1 (13.5)	114.3 (12.9)	0.69
Diastolic blood pressure, mmHg	68.1 (9.3)	68.0 (9.6)	68.1 (9.2)	0.90

GDM = gestational diabetes mellitus.

BMI = Body mass index.

OGTT = oral glucose tolerance test.

### Data safety and monitoring plan

This trial is monitored by an independent Data and Safety Monitoring Board (DSMB). The DSMB evaluates the progress of the trial with biannual assessments of participant recruitment, accrual and retention. We have restricted our EMR-based assessment of adverse events to those that could be related to the intervention (such as ankle sprains, etc.), or serious events that required hospitalization or a visit to the emergency department. For women who are no longer members of the health plan, adverse events are assessed via survey.

### Evaluation of the intervention

To understand and inform the intervention’s implementation and potential for adoption by the health system [10] we conducted semi-structured interviews with KPNC clinical and operational leaders before the start of the study and at the end. At the end of the early postpartum phase, we will also conduct semi-structured interviews with the lifestyle coaches. Satisfaction with the intervention is assessed using a mailed questionnaire at approximately 8 months postpartum among women who completed at least one telephone session.

Data on direct medical costs of the intervention, which include all costs associated with implementing and maintaining the intervention (excluding the cost of evaluation), and all medical service costs, are collected through the EMR and surveys. In addition, at each survey, we ask participants if they were still KPNC members and if they have had any medical visits outside KPNC. We also assess perceived health status via survey with the EQ-5D [35].

### Statistical analyses, power and sample size considerations

All analyses comparing the effectiveness of each intervention strategy on our primary and secondary outcomes will be by intent-to-treat, including all eligible study subjects with measurements at all available time points, as appropriate to the outcome; analysis of treatment differences will be by original treatment condition assignment, regardless of adherence. The two primary outcomes for this study are the achievement of the postpartum weight goals and change in weight. Analyses will utilize marginal regression models for estimation of population average intervention effects, with linear mixed regression in analyses of weight change and logistic regression with estimation via generalized estimating equations (GEE) in analyses of reaching the weight goal. These regression models account for the within medical facility correlation between patients and within person correlation among repeated measurements, for valid estimation of treatment effects and associated standard errors. Variation in treatment group differences over time will be examined via the introduction of appropriate cross-product (interaction) terms between groups and time.

At the planning stage of this study, we expected a total sample size of 2,432 women diagnosed with GDM in a given year at the 44 facilities, and that approximately 76% would have weight measured at/near 12 months postpartum. Minimum detectable differences in the proportion meeting weight goals ranged from 0.060 to 0.10, across the range in expected proportion meeting goal in the usual care condition (0.15 - 0.25) and across the range in expected intraclass correlation (0.01 - 0.05) [chi-square test for two proportions, two-sided test, α = .05, power = .80]. For our actual sample size of 2,320 eligible women with GDM at the 44 facilities and with an expected 70% with weight measured at or near 12 months, the minimum detectable difference in proportions ranges from 0.062 to 0.11. The minimum detectable difference in mean weight at 12 months postpartum ranged from .16 to .23 standard deviation units at the planning stage, with no change in detectable effect sizes with the slightly lower actual sample size and proportion with weight measured at or near 12 months. These calculations are conservative, given that primary analyses will utilize multiple weight measures per person via mixed effects regression analyses.

The human subjects committee of the Kaiser Foundation Research Institute waived the requirement for individual informed consent for the intervention component, given the pragmatic, cluster randomized design [38,39]. For the survey component, women give verbal consent to participate via telephone. Women attending medical facilities randomized to the intervention can opt out of the lifestyle program at any time and individuals can also refuse participation in the study surveys or postpartum health check-ups at any time. All intervention and data assessment materials for the GEM study have been approved by the human subjects committee of the Kaiser Foundation Research Institute; the trial is also registered at clinicaltrials.gov (NCT01344278).

## Discussion

The GEM study will test the comparative effectiveness of diabetes prevention strategies for women with GDM in a real-world clinical setting. Women with GDM represent a population at high risk for diabetes and are identified by the health care system at a very young age, due to a standard of prenatal care that includes screening for GDM. Since fifty percent of women with GDM develop diabetes within 5 years after delivery [40], diabetes prevention programs should be initiated in the perinatal period.

As highlighted recently [41], there is a need for evidence regarding the effectiveness of lifestyle modification for the prevention of diabetes in women with GDM, as well as confirmation that a diabetes prevention program delivered at the health system level is able to successfully reach this population. Given the ethnic disparities in GDM, there is a strong need to develop interventions that can reach diverse populations in real world settings to ensure that any impact found is generalizable [41]. Most (73%) of our GDM sample is ethnic minorities, including Asians, Hispanics, and African-Americans, thereby enabling our study to generate findings which are generalizable to women from ethnic groups at high risk for diabetes. In recent years, the U.S. has made significant investments in comparative effectiveness research to provide scientific evidence regarding the effectiveness of strategies for preventing, diagnosing, treating and managing medical conditions in clinical settings. To discover which strategies work best in a clinical setting, pragmatic randomized trials may provide a more robust approach than traditional randomized trials because they are not conducted among a specifically screened and/or select volunteer patient population and occur within routine clinical practice settings [42]. Our cluster randomized trial incorporates several key characteristics of pragmatic trials, such as a non-selective population of women with GDM (given that 96% of pregnant women are screened for GDM at KPNC), the broad inclusion of patients with GDM (i.e., no exclusions based on other medical conditions or language), an intervention delivered at the health system level, and the use of readily available clinical characteristics and outcomes in the EMR.

Given the diverse racial/ethnic composition of women in the GEM study and the use of a case management telephonic health model to deliver the intervention, our DPP-derived intervention has great potential to be adopted and/or translated into other clinical settings. In addition, the intervention uses motivational interviewing techniques to promote changes in diet and physical activity, thereby enhancing patient-centered communication in a healthcare setting. We expect the information obtained from this trial will be used to inform future pregnancy and postpartum health practices for women with GDM, and develop public health policies related to the prevention of diabetes in this population of young women at high risk for diabetes.

## Competing interests

The authors declare that they have no competing interests.

## Authors’ contributions

AF, MMH, CLA, and CPQ designed the study; AF, CLA, SDB, YC, and JC developed and implemented the intervention; AF, SFE and AAM researched the data; AF and BJE directed the diet assessment; AF, BS, and SFE directed the physical activity assessment; AF, YC and EPG directed the infant feeding assessment; AF, NPG and JAS directed the program evaluation; and AF, CPQ and AT analyzed the data. AF wrote the manuscript and the other authors reviewed/edited the manuscript and contributed to discussion; AF is the guarantor. All authors read and approved the final manuscript.

## Pre-publication history

The pre-publication history for this paper can be accessed here:

http://www.biomedcentral.com/1471-2393/14/21/prepub
